# The patient experience of radiotherapy for breast cancer: A qualitative investigation as part of the SuPPORT 4 All study

**DOI:** 10.1016/j.radi.2020.09.011

**Published:** 2021-05

**Authors:** H. Probst, K. Rosbottom, H. Crank, A. Stanton, H. Reed

**Affiliations:** Sheffield Hallam University, UK

**Keywords:** Breast cancer, Radiotherapy, Patient experiences, Patient support

## Abstract

**Introduction:**

Breast cancer is a global health problem with 2.09 million cases of breast cancer diagnosed worldwide in 2018. With an increase in breast cancer survival attention has now focussed on the impact treatment side effects can have on the quality of life for women during survivorship. The aim of the SuPPORT 4 All project is to develop a support bra for use during radiotherapy, that can reduce normal tissue toxicity (for women with larger breasts) and provide accuracy, dignity and modesty for all women. The first stage of the project involved a co-design process to understand the current patient experience where no support bra or modesty device is used.

**Method:**

A participatory co-design methodology was adopted. Workshops were held with patient representatives (n = 9) to seek understanding of experience during radiotherapy; a total of three workshops over 4 h. The workshops were audio recorded and framework analysis was adopted to identify key patient experiences.

**Results:**

Twelve categories and twenty-six sub categories were identified specific to patient experience. Patient concerns focussed on information provision, Healthcare Practitioner (HCP) knowledge of breast lymphoedema, lack of choice, experiences of being naked, and feelings of disempowerment.

**Conclusions:**

A number of areas were identified that had negative effects on overall patient experience.

**Implications for practice:**

Practitioners should consider patient dignity when configuring services to support patient needs regarding undressing, outside or inside the linear accelerator room. Additionally, practitioners should have an understanding of the impact permanent tattoos may have on some patients’ wellbeing and the impact that breast lymphoedema has on patient quality of life. Practitioners should also consider methods to encourage patient empowerment during radiotherapy; supporting patient self-monitoring of side-effects may be one way to facilitate this.

## Introduction

Breast cancer is a global health problem with 2.09 million cases of breast cancer diagnosed worldwide in 2018,^1^ accounting for 11.6% of all cancer incidence. Survival from breast cancer has improved in many countries, with over 1 million women surviving the disease in 2012.^2^ Hence as more women survive, attention has now focussed on the impact treatment related side effects and treatment experiences can have on the quality of life for women and their ability to cope during survivorship.

There has been significant research in the field of breast irradiation techniques over recent years to reduce treatment related sequela.^3^^,^^4^ The experience of involvement in clinical trials such as HeartSpare (I and II)^5^^,^^6^ and the IMPORT trials^7^^,^ means many radiotherapy departments are moving to more complex radiotherapy techniques including Intensity Modulated Radiotherapy (IMRT), and the use of breath hold techniques, or implementing simultaneous integrated boost techniques; some of these techniques enable greater lung and heart sparing.

However, there has been less research investigating patients' experiences of the delivery of radiotherapy. There is a range of research that focusses on patient's lived experiences of a breast cancer diagnosis, perceptions of treatment and experiences of survivorship or assessments of symptoms from radiotherapy.^8^, ^9^, ^10^, ^11^, ^12^, ^13^ However, the research tends to focus primarily on information needs, or frequency or experience of side effects. There is little focus on the experience of attending for breast irradiation itself.

Schnur et al. (2009) reported on the assessment of patient diary reflections written during a course of breast irradiation (14) (n = 15 women). The key themes identified in this qualitative study focussed on timing (for example timing of side effects), health after treatment, self-esteem and the mysteriousness of radiotherapy and how it works.^14^ However, there was no indication or discussion of the impact permanent tattoos may have on patient experience and limited discussion of the impact wearing gowns or being naked might have on patients’ overall treatment experience.

For example, in the majority of radiotherapy centres worldwide women lie for breast irradiation bare from the waist upwards, with up to four therapy radiographers (Radiation Therapists) including students and men adjusting and manipulating their thorax and breast in preparation for treatment. Many women are known to have body image concerns following breast surgery^15^^,^^16^ and the manipulation required to position the breast for treatment can be undignified. In one study body image scores measured using the four body image questions from the European Organisation for Research and Treatment for Cancer questionnaire (EORTC QLQ Br23) identified patients on active treatment (radiotherapy or chemotherapy) may have significantly worse body image than patients that are post-treatment (6 months post treatment) or women in the general population without a history of breast cancer.^16^ Body image scores were 47.5 vs 53.4 and 70.2 for those on active treatment, post-treatment and controls respectively, where higher scores reflect a more positive body image. Hence, patients may be at their most vulnerable in terms of low body image when they attend for radiotherapy, and at a time when they are asked to lie naked in front of healthcare workers.

In addition, most radiotherapy centres rely on the use of at least three permanent tattoos (but it can be more) marked on the patient; the use of permanent tattoos can be a concern to some patients^17^ and may have an impact on body image.^18^ In addition, a range of practices occur across radiotherapy centres in terms of physical undress in waiting rooms and in the linear accelerator room itself. The use of gowns or how undressing is managed^19^ can vary, with potential effects on dignity and modesty or emotional experiences.^20^

The primary aim of the SuPPORT 4 All project was to design, produce and test a support bra for women undergoing breast irradiation. In the development stage of the project we aimed to determine current experiences of radiotherapy for breast cancer. Specifically, we wanted to determine answers to the following questions:

1. What are women's experiences of the radiotherapy pathway from radiation planning to completion of a full treatment course?
2. What are women's views of being naked (from the waist upwards) during planning and treatment sessions?
3. How do permanent tattoos affect the overall patient experience?

These questions were relevant to how we approached the design of a bra support device.

## Methodology and method

**Figure 1:**
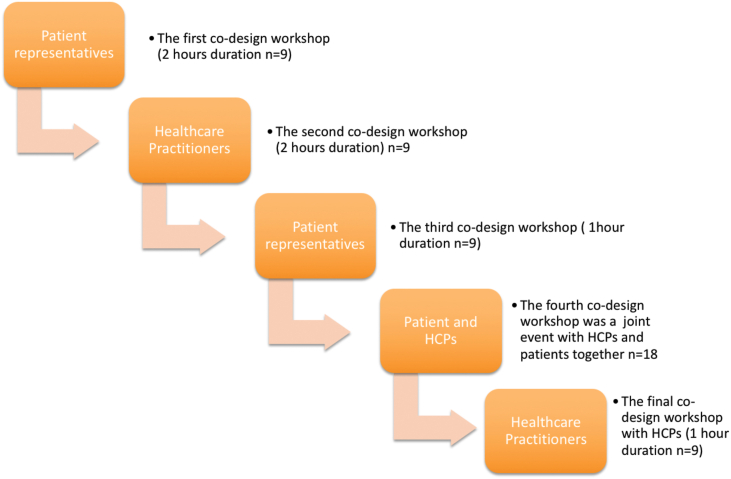
A diagrammatic presentation of the Co-Design process.

The Medical Research Council (MRC) framework for complex interventions^21^ was adopted for the SuPPORT 4 All study; this stage of the study presented here represents the development phase of the MRC framework. To gather stakeholder insights, we designed a series of workshops (five in total) (Fig. 1). A participatory co-design method^22^ was adopted as this method offered the opportunity for co-interpretation of design refinements by the researchers and the eventual users of the bra; both patients and healthcare practitioners, specifically therapy radiographers (also known as Radiation Therapists or RTTs), and physicists or dosimetrists. The participatory co-design approach ensures participants’ interpretations are embodied in the developing design and that the resultant product is shaped through a joint (researcher and participants) vision.

The participatory co-design process involved three key elements:

1. Understanding the process and the patient experience.
2. Clarification of user goals and expectations of the final product and
3. Iterative shaping of the bra technology detail.

The focus of the results presented here is on understanding the process and the patient experience (element 1) above.

### Recruitment and selection of subjects

Sampling was purposive; we were keen to discuss experiences of breast irradiation with women that had undergone radiotherapy to the breast within the last five years. We approached a local support group for cancer patients to request assistance with reaching individuals that met the following criteria:

• Diagnosed with breast cancer,
• Undergone conservative surgery (wide local excision leaving an intact breast), and
• Had experienced radiotherapy as part of the treatment package.

The local support centre invited us to present details of the study to centre staff, who agreed to mention the study to relevant individuals who attended the centre. We also had individuals that volunteered to join the co-design workshops as they had heard about our study through other contacts. All participants signed a consent form prior to attending. As the design of the support bra was to be discussed in the workshops all participants were also asked to sign a confidential non-disclosure agreement to protect details of the bra design.

Details of the setting, data collection process, workshop moderators and facilitators can be found in the Supplementary material (S1)

### Pre-workshop preparation

**Figure 2:**
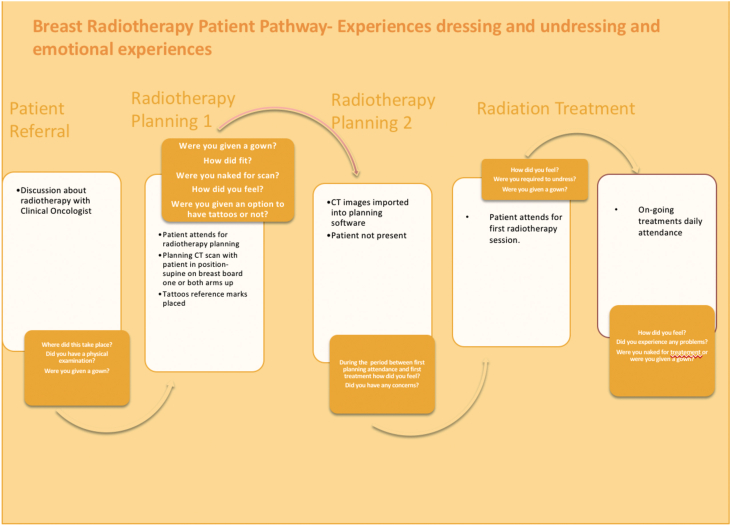
Pre-workshop preparation, the radiotherapy pathway participants were asked to annotate.

Prior to attendance participants were sent a link to a Cancer Research UK short video on breast radiotherapy^23^; this served to simply remind participants of their own radiotherapy experience. In addition, participants were also asked to annotate a diagrammatic presentation of the radiotherapy pathway (see Fig. 2) with details of their experiences at each point in the pathway. It was explained to participants that these would be discussed in the first co-design workshop.

### Data analysis

The transcribed data was imported into NVivo software (version10) and analysed independently by A1 and A2 using framework analysis. Framework analysis is a well-used technique for organising and charting qualitative data in health research.^24^ The researchers familiarised themselves with the data by reading and re-reading the transcripts and playing back the audio recordings. Coding followed a line by line open coding process. A set of codes were identified by each researcher (A1, A2) from workshop 1 and compared. Discussion of the two independent code lists led to a single analytical framework from which a set of themes and sub themes were derived.

### Minimising opportunities for researcher bias

A number of techniques were used to minimise opportunities for bias (see Supplementary materials 1 for more detail). It was important the research findings were regulated through the participant's lens,^25^ as this is central to the co-design methodology. Member-checking was used within and after the workshops to ensure the participants' voice (not the researchers) was presented.^26^ Member-checking is a process of confirming that data analysis has captured the participants original intended meaning and is important for ensuring credibility of the outcomes.

### Ethics approval

The Health and Social Care research ethics committee of the Host Institution gave ethical approval for the study; all participants were recruited outside the National Health Service (NHS).

### Findings

Nine patient representatives attended the first, third and fourth co-design workshops (see Fig. 1). Of those participants that consented to participate none dropped out of the study. Time since radiotherapy for each participant ranged from less than one year to five years. Across all workshops (workshop one to five in Fig. 1) the first data analysis by A1 and A2 independently identified 131 separate codes. Following discussion these codes were refined and condensed to fourteen categories and ninety-six sub-categories in total. Table 1 identifies the twelve categories and twenty-six sub-categories related specifically to patient experience of radiotherapy; the remaining two categories were feedback on the bra design and challenges to delivering breast irradiation from HCPs.

**Table 1:** Categories and codes specific to patient experience of the radiotherapy journey.

Categories identified from user discussions	Sub Categories
Information needs	Knowledge of Treatment Mis-information Timing of Information Preconceptions of RT
Exposure	Issues of Modesty Wearing a gown in a public place
Emotional experience	Feeling embarrassed
Interactions with HCPs	Staff attitudes Feeling a burden
Finding your voice	Being listened to Disempowered
Technology focused care	Impersonal Systems and processes-including having confidence in staff and the process
Choice	Communication issues Feeling oppressed Having to have tattoos
Fear	Fear about treatment accuracy, minimising errors Feeling frightened
The waiting room experience	Inappropriate entertainment
Getting to radiotherapy	Physically getting there to the radiotherapy centre The emotional journey-what has come before (including chemotherapy, surgery the end of a long process)
Impact of side effects	Skin reactions Finding a comfortable bra to wear during the radiotherapy period
The changed self	Lost self confidence Change to personal image/body image Wanting to feel normal

The findings are reported according to the three principle questions driving the stakeholder engagement workshops:

1. What are women's experiences of the radiotherapy pathway from radiation planning to completion of a full treatment course?
2. What are women's views of being naked (from the waist upwards) during planning and treatment sessions?
3. How do permanent tattoos affect the overall patient experience?

### Women's experiences of the radiotherapy pathway

#### Information needs

Participants’ discussions highlighted some deficiencies in the provision of information that affected the overall patient experience.

**Figure 3:**
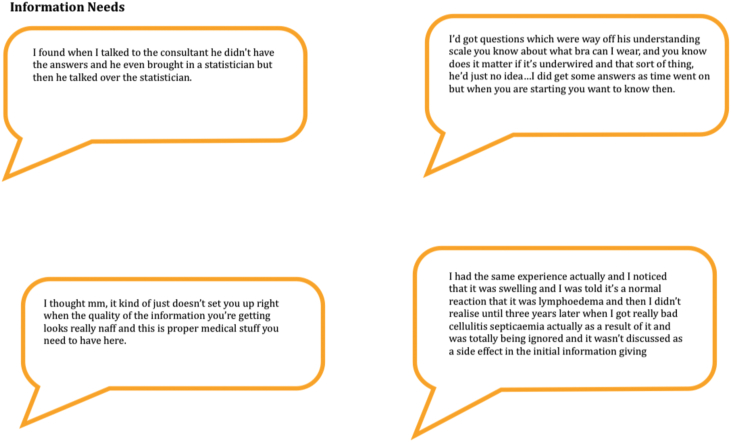
Information needs.

Some participants had specific questions that were not answered (see Fig. 3 and the Supplementary material S2.1.1)

There seemed to be a lack of personalisation of information that was provided and “*it just seemed to be that this is the protocol; this is what we're doing*”

The timing of information was important for participants; one reported receiving an information leaflet containing all the details about treatment side effects after she had signed the consent form (rather than before) and she became alarmed at the potential side effects having already signed and given her consent to proceed. While another participant, received an information leaflet that had been photocopied and the quality was poor, the images were difficult to see and the words were a bit “*wonky*”.

Many of the study participants had developed breast lymphoedema during or after radiotherapy and most were unprepared for this happening (Fig. 3).

#### Emotional experience

**Figure 4:**
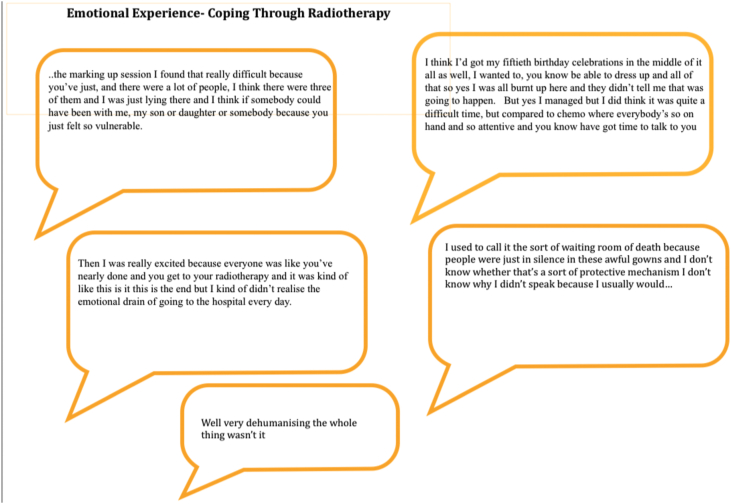
Emotional experiences-coping through radiotherapy.

Participants commented on the emotional impact of attending for radiotherapy. Often radiotherapy is seen and spoken of as the easy part of the breast cancer treatment pathway; often promoted by radiographers as ‘the easy bit’. In fact, participants found their radiotherapy experience difficult in ways they were not expecting. In their words they found it ‘impersonal’, and ‘dehumanising’, they felt ‘vulnerable’ and ‘disempowered’ (see Fig. 4).

### Women's views of being naked

#### Exposure

**Figure 5:**
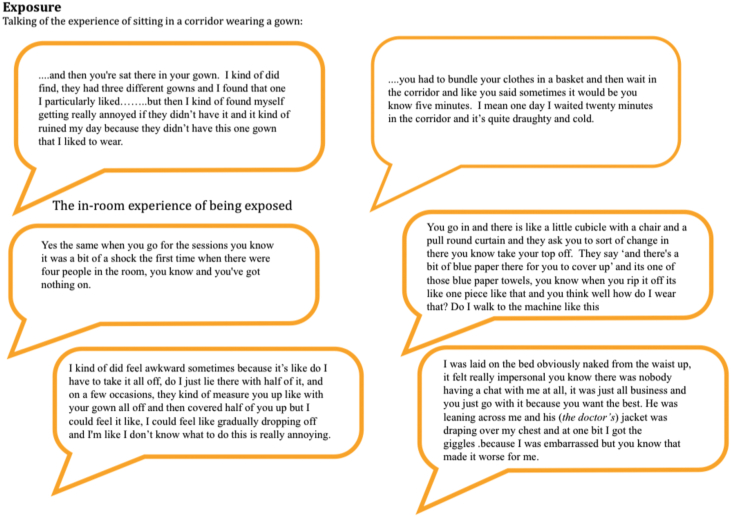
Exposure.

Discussions about being exposed included experiences both inside and outside of the linear accelerator room. Participants commented about the negative experience of having to wait in draughty corridors dressed in a hospital gown as well as the experience of being undressed while in the treatment room. There was also comment about how wearing a gown affected the dynamics of the waiting room experience, how easy or difficult it was to make conversation while wearing a gown and not your normal clothes (see Fig. 5).

#### Modesty and dignity

**Figure 6:**
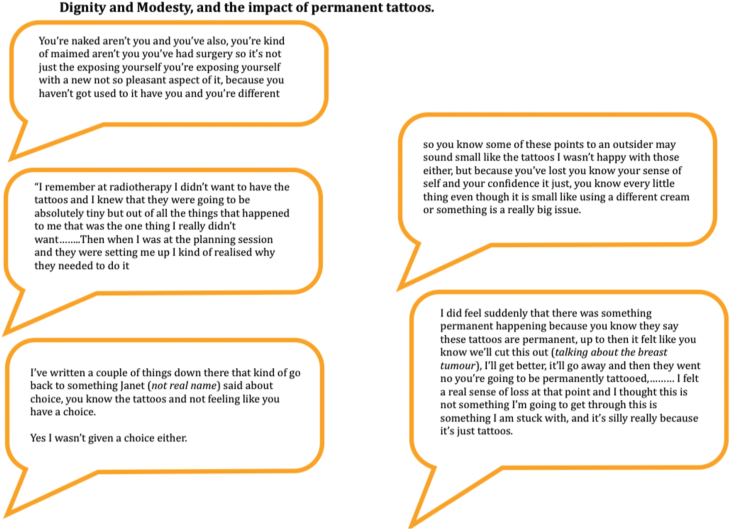
Dignity and modesty, and the impact of permanent tattoos.

While patients understood the need for accuracy and achieving the best outcomes, they struggled to maintain dignity throughout the radiotherapy process. Experiences of waiting for treatment in waiting rooms and the need to be quick once in the treatment room meant patients were either required to sit in the waiting room in a gown (without a bra) or wear their own clothes, but for speed go braless (Fig. 6).

There were also comments about the number of people in the room during planning and treatment sessions and the knowledge that teaching was going on with lots of people present and this added to the feelings of vulnerability lack of modesty and difficulty to maintain dignity (Fig. 5). Feelings of loss of dignity were also compounded by a sense of loss of the ‘self’; the person they used to be, and the control that they used to have over their lives.

*“I felt stripped of myself somehow and really disempowered.” (S2.1.2)*

### The impact of tattoos on the patient experience

Tattoos are currently seen as the gold standard for aligning patients for breast irradiation. Women in our stakeholder workshops reported a range of different experiences, emotions and knowledge about the use of tattoos, and whether they had a choice to have them or not. While a few women didn't mind having the tattoos *“it's fine it's nothing”.* Others had often-profound experiences that were linked to other experiences in the radiotherapy journey such as lack of choice, disempowerment and realisation that what they had was not a small insignificant disease but something bigger; something that required a permanent mark as a reminder and marker of the enormity of the cancer condition.

## Discussion

The results highlighted some agreement with previous research but also identified some new insights on the patient experience of the breast cancer radiotherapy pathway; these are discussed under the key areas of inquiry.

### Women's experiences of the radiotherapy pathwa*y*

Provision of information has been shown to decrease emotional distress, and enhance self-care strategies.^27^ Long (2001) in a qualitative study of twenty patients with different cancer diagnoses identified the importance of information provision in giving patients a sense of control and was significant in a patient's ability to cope during radiotherapy. Feeling prepared was important for individuals in Long's study as was the timing of information and this was identified in our study also. Seeking information has been shown to be a coping mechanism particularly for younger breast cancer patients.^28^

Schnur et al. (2009) studied women's experiences (n = 15) of radiotherapy through personal diaries.^14^ Four key themes were highlighted; timing was one theme. However, unlike the present study timing was primarily related to when symptoms occurred rather than timing of information. Yet Schnur et al. (2009) conclude that the way information is presented to patients about when symptoms are likely to start (and end) maybe critical for how patients subsequently react to symptoms as they occur.

A clear message emerged from half of the workshop participants that breast lymphoedema was poorly communicated to them, and getting their concerns heard by HCPs was problematic. Breast lymphoedema appears silenced in the literature with an abundance of research on arm lymphoedema^29^^,^^30^ but very limited research on breast lymphoedema^31^; focusing on quantifying incidence with little emphasis on understanding the patient experience.

#### Emotional experience

Women commented on the emotional ‘drain’ associated with attending for radiotherapy and how ‘dehumanising’ the whole process was. These words reflect the whole process including waiting room experiences, in-room linear accelerator (Linac) experiences and interactions with healthcare staff. Mose et al. (2001)^32^ identified a significant correlation between these external factors (waiting room, linac room perceptions) and radiotherapy associated anxiety (p < 0.02) and while they do not reflect causation it is reasonable to assume that good waiting room and Linac room experiences may positively reduce radiotherapy associated anxiety. The experience of lying alone on a CT scanner or Linac couch while HCPs focus on set-up parameters and patient alignment may exacerbate patient feelings of technology-focussed rather than patient focussed care during planning and treatment procedures; this contrasts with the human contact and attention received during chemotherapy.

### The experience of being naked following breast conserving surgery

It is known that women who have undergone breast surgery experience issues with altered body image.^15^^,^^33^ What is less clear is the impact of having to lie naked on a bed from the waist upwards when already experiencing an altered body image, or wearing a gown in public waiting areas.

#### Exposure

The experience of lying naked for radiotherapy treatment is rarely discussed in the research literature; it remains the domain of personal blogs from cancer survivors. Yet it was recognised twenty years ago as being problematic for women.^19^ Harris and Haas (1997) developed a modesty gown for women to wear during breast irradiation (the Plymouth Gown), during evaluation (n = 20) seventy percent of women indicated feeling self-conscious about their body at the time; 86% preferred using the gown for treatment.

#### Modesty and dignity

Despite the initial success of the Plymouth gown uptake across radiotherapy centres has been limited; due in part to differences across centres in the use of positioning marks. Some women have reported that the gown identifies them in waiting rooms as a breast cancer patient; while other patients sit in their own clothes indistinguishable from carers.^20^ Women in our study found the use of a gown to partially cover them during treatment exposure (providing modesty for the unaffected breast) was often distracting and led to some anxiety when the gown started to slip. Apart from these two studies on the breast gown^19^^,^^20^ attention to the experience of women lying naked for breast irradiation is absent in the literature.

### Use of permanent tattoos

We are aware from previous research^17^ that there are mixed views about the use of permanent tattoos for breast radiotherapy positioning. There are currently limited skin marking alternatives to the use of permanent black tattoos; although the use of ultra-violet tattoos provide a possible option for some women.^18^ However, in the study by Landeg et al. (2016) the UV tattoos were not successful on sub-Saharan skin tones. There was some indication that body image post radiotherapy maybe enhanced as a result of using UV tattoos (56% of those with UV tattoos reported improvements in body image score at one month, compared with only 14% in the black ink tattoo group). However, median body image scores were consistent from baseline to one-month post treatment for the UV tattoo patients, improving by only 1.0 by six months. In the black ink tattoo group median body image scores from baseline to six months increased by only 0.5; differences in median scores at six months was not statistically significant.^18^

There is currently limited clinical experience of using UV tattoos for breast irradiation and for some patients the fact they are still permanent and will be visible under black lighting conditions, may make them an unattractive option. For the women in our study the issue surrounding the use of permanent tattoos was closely linked to a lack of choice. Principally participants felt they had little or no option when it came to having permanent tattoos; despite evidence that careful use of semi-permanent ink (for those that prefer this option) produces equivalent random and systematic errors.^17^

### Study limitations

The research presented here is from a small sample of women, it is not intended to be reflective of the experiences of breast irradiation for all women. However, it does provide insight in to the experiences of a few and to highlight areas of practice where HCPs can make small improvements that could enhance experience.

## Conclusions and next steps

This small scale participatory co-design study highlighted a number of areas where it may be possible to improve the patient experience of radiotherapy to the breast. It has confirmed the importance of good quality information and attention to the timing of information giving. Specifically, this study has identified potential gaps in HCP knowledge and understanding of breast lymphoedema, added further concern about the impact of using permanent black tattoos and insight in to patient dignity regarding undressing and exposure outside or inside the linear accelerator room. This study has highlighted a number of areas of new knowledge including patient experiences of disempowerment that can hamper opportunities for a fast recovery and return to normal activities or work. Opportunities for patients to be involved in self-monitoring of symptoms may reduce these experiences. To this end we have co-designed two self-monitoring tools for patients to complete during radiotherapy to monitor skin changes and the development changes in the breast that may be indicative of breast lymphoedema.

## Data Sharing

Due to the consent process for participants the qualitative research data is not available for sharing.

## Funding source

This work was supported by funding from the 10.13039/501100000272National Institute for Health Research Invention 4 Innovation Fund (funding reference II-LA-0214-20001). The funding body had no role in the design, analysis or write up of this research.

## Ethics statement

Ethics approval for this stage of the study was provided by the Health and Social Care, Research ethics sub-committee of Sheffield Hallam University (The Host Institution). All participants were recruited outside of the NHS.

## Conflict of interest statement

Heidi Probst, Heath Reed and Andrew Stanton are named on the UK and European patents for the Support 4 All bra. The design of the Support 4 All bra was refined as part of a programme of research funded by the NIHR (please see below for funding details). The work presented in this manuscript formed part of this programme of research and was the co-design stage of workstream 1 of the NIHR i4i funded research. The authors have no other conflicts of interest to declare.
