# Supplementary material for: The patient experience of radiotherapy for breast cancer: A qualitative investigation as part of the SuPPORT 4 All study
Source: Radiography (Lond). 2021 May;27(2):352–9. doi: 10.1016/j.radi.2020.09.011 (PMC8063584; doi:10.1016/j.radi.2020.09.011)
Supplement: Multimedia component 2 [file mmc2.pdf]

Supplementary Material

S1 Additional method details

Recruitment and Selection process

All individuals that showed an interest in the study were asked to leave their contact details and a member of the project team (A2) made personal contact with each volunteer (either by email or by telephone) sending further information and details of the workshop dates. Volunteers were given time to read all the information sent to them before confirming attendance at the first co-design workshop.

Setting and Data Collection Process

All co-design workshops took place on the University campus of the Host Institution. The patient representatives attended three workshops over two separate days. The first workshop lasted for two hours, the basic format for the first workshop can be found below. During the first workshop an introduction was given about the project team (their experience, background and roles) and the project itself; including how the project started, where the idea for the bra stemmed from, work that had been completed up to that point as well as the purpose of the co-design workshops. While there was a structure to the workshop, questioning was open and led by the participants' accounts of their radiotherapy experience. Pre-determined questions were only used to elicit information about the bra design that was required for bra refinement, and only where the topics had not already been covered by participants' discussions.

The second workshop was split into two halves, each an hour long. The first half was used to feedback to participants the changes made to the prototype bra based on comments made during workshop 1 and further feedback on the prototype was sought at this stage. The second half was a joint co-design workshop including members of the HCP group. In this workshop participants were divided into two groups with an equal mix of patient representatives and HCPs in each group. In this co-design workshop participants were given a range of resources and tasked with formulating two patient self-reporting tools; the new tools were based on comments and concerns from patients and HCPs raised in earlier workshops (1 and 2 in Figure 1).

All workshops were audio recorded and transcribed verbatim after each event, and A2, A3, and A5 also took notes to support transcribed data.

Workshop moderators and facilitators

Other than the participants, four members of the project team attended each co-design workshop (A1, A2, A5, A3). The workshop lead was A1 on all occasions, supported by A2 (who took notes). The design team (A5 and A4) attended each workshop in order to gain in-depth understanding of issues of importance to potential users and to facilitate any design discussions. A1 (project lead) is an academic therapeutic radiographer with fourteen years experience of clinical practice as a therapeutic radiographer in the NHS and eighteen years experience as a researcher in academia (with experience of leading and analysing qualitative research). A2 had made contact with all patient representatives prior to the co-design workshops, is a qualified therapeutic radiographer, now a lecturer and novice researcher, but with clinical experience in the NHS. A1 and A2 were not involved in clinical delivery of radiotherapy during the periods when any of the participants may have undergone treatment.

Minimising Opportunities for Researcher Bias

In qualitative research it is acknowledged that researchers cannot be divorced from previous experiences that may influence subsequent interpretations of the research data they become immersed in. Therefore, both A1 and A2 wrote reflexive accounts prior to conducting the workshops and the subsequent data analysis. In addition, the research findings were regulated through the participant's lens[25], as this is central to the co-design methodology. To assess how accurate participant's views were represented in the analysis member checking was undertaken both during, and after the workshop. Within the workshop A1 used summarising at key points to clarify patient experiences being reported. In addition, at the end of the first session patients were asked to write a summary message together that represented a key take home message they wanted healthcare practitioners to hear. After the workshops attendees were sent a copy of the developing analytic categories and asked to comment on how accurate these represented their radiotherapy experience and the discussions from the two hour event[26]. This approach provided participants with an opportunity to correct or challenge what may be perceived as mis-interpretations of the discussion, as well as an opportunity for participants to proffer further information they may have omitted to provide in the workshop.

Workshop 1 Schedule

Workshop 1 Schedule

- Pre-workshop refreshments for participants to mingle and meet other workshop members and A1 and A2 from the project team informally.
- Session start, section 1- Brief introduction to the members of the project team in attendance, led by A1. Discussion of patient experiences. Patients each given time to discuss and present their own experience.
- Section 2- Free discussion, A2 summarised key points on flip chart paper for constant member-checking.

- Section 3- Product samples and design images circulated, prototype bra used as prop to explain proposed use.
- Section 4-A summary statement from the group members was developed about the key issues for patients having radiotherapy for breast cancer. The summary statement was written on flip chart paper to display in the HCP co-design workshop as a reminder to practitioners of the concerns faced by patients.
- Section 5- a set of specific questions to gain feedback on the proposed bra design were discussed for example, is there a preferred colour?
- Final round up-bringing participants back to the 'here and now', refreshments were available to aid informal chat prior to session end.

Additional discussion of potential study limitations and Strengths.

A further potential limitation of the study is that participants were recruited from a local support centre. Could these individuals be more likely to have had poor experiences, compared with individuals who do not attend support services? Women with breast cancer seek and find support from many different sources including:

- Existing social networks, friends, and family, or work colleagues.
- Online forums such as the Breast Cancer Now forums.
- Social Media (Twitter, Facebook groups)
- Charity centres and local support centres.

Attendance either in person or virtual (on an online forum) for support can be for a variety of reasons. It might be for support with hormone side-effects, support with helping their children to cope with their cancer diagnosis, help with getting back to exercise, attendance at healthy eating programmes or use of complementary therapies, or wig services. Many support centres run healthy living programmes for breast cancer patients to support living well with and beyond cancer. In addition, many women attend groups at support centres to give something back to other women going through cancer treatment. Furthermore, not all of the participants in this study were recruited from the local support centre providing opportunity for a range of experiences to be heard.

Despite the study limitations presented in the main article and above, the research design facilitated rich data on patient experiences of breast irradiation that have not been previously studied such as loss of personal control or disempowerment; identifying important new areas for further service development and research.

S2 Additional patient quotes in support of identified themes.

S2.1 Women's experience of the radiotherapy pathway

S2.1.1 Information needs

"I found when I talked to the consultant he didn't have the answers and he even brought in a statistician but then he talked over the statistician."

There seemed to be a lack of personalisation of information that was provided and "it just seemed to be that this is the protocol; this is what we're doing"

S2.1.2 Emotional experience

"I think I'd got my fiftieth birthday celebrations in the middle of it all as well, I wanted to, you know be able to dress up and all of that so yes I was all burnt up here and they didn't tell me that was going to happen.   But yes I managed but I did think it was quite a difficult time, but compared to chemo where everybody's so on hand and so attentive and you know have got time to talk to you."

"Then I was really excited because everyone was like you've nearly done and you get to your radiotherapy and it was kind of like this is it this is the end but I kind of didn't realise the emotional drain of going to the hospital every day."

"I used to call it the sort of waiting room of death because people were just in silence in these awful gowns and I don't know whether that's a sort of protective mechanism I don't know why I didn't speak because I usually would..."

"Well very dehumanising the whole thing wasn't it.."

"I was laid on the bed obviously naked from the waist up, it felt really impersonal.."

"I felt stripped of myself somehow and really disempowered………. by that point I felt completely, I had no power over my own life at all,"

"but I think it's also a very disempowering process and it needn't be that disempowering, because I never found the surgery disempowering in the same way,"

"I mean when you've had a, particular you know when you've had a professional life where you've been making the decisions, you've been controlling things and suddenly you have no control you're just doing as you're told that was difficult"

"It's just made me aware actually of how passive I was…"

S2. 2. Women's views of being naked

S2.2.1. Exposure

"….and then you're sat there in your gown. I kind of did find, they had three different gowns and I found that one I particularly liked……..but then I kind of found myself getting really annoyed if they didn't have it and it kind of ruined my day because they didn't have this one gown that I liked to wear."

S2.2.1.1 The in-room experience of being exposed.

“Yes the same when you go for the sessions you know it was a bit of a shock the first time when there were four people in the room, you know and you've got nothing on.”

“….you go in and there is like a little cubicle with a chair and a pull round curtain and they ask you to sort of change in there you know take your top off. They say ‘and there's a bit of blue paper there for you to cover up’ and its one of those blue paper towels, you know when you rip it off its like one piece like that and you think well how do I wear that? Do I walk to the machine like this?”

“I kind of did feel awkward sometimes because it’s like do I have to take it all off, do I just lie there with half of it, and on a few occasions they kind of measure you up like with your gown all off and then covered half of you up but I could feel it like, I could feel like gradually dropping off and I'm like I don’t know what to do this is really annoying.”

“I was laid on the bed obviously naked from the waist up, it felt really impersonal you know there was nobody having a chat with me at all, it was just all business and you just go with it because you want the best. He was leaning across me and his (*the doctor’s*) jacket was draping over my chest and at one bit I got the giggles because I was embarrassed but you know that made it worse for me.”

S2.2.2. Modesty and dignity

“Yes the same when you go for the sessions you know it was a bit of a shock the first time when there were four people in the room, you know and you've got nothing on.”

S2.2.2.1.The impact of tattoos on the patient experience

“I’ve written a couple of things down there that kind of go back to something Janet said about choice, you know the tattoos and not feeling like you have a choice.”
“Yes I wasn’t given a choice either.”

“I did feel suddenly that there was something permanent happening because you know they say these tattoos are permanent, up to then it felt like you know we’ll cut this out (*talking about the breast tumour*), I’ll get better, it’ll go away and then they went no you’re going to be permanently tattooed,……… I felt a real sense of loss at that point and I thought this is not something I’m going to get through this is something I am stuck with, and it’s silly really because it’s just tattoos.”
